# A literature review of the regional implementation of the central Swedish government’s health care reforms on choice and privatization

**DOI:** 10.1186/s13561-015-0076-0

**Published:** 2015-12-09

**Authors:** Björn Ekman, Jens Wilkens

**Affiliations:** Division of Social Medicine and Global Health (SMGH), Department of Clinical Sciences, Lund University, Jan Waldenströms Gata 35, 205 02 Malmö, Sweden

**Keywords:** Policy reform, Political economy, Specialized health care, Sweden

## Abstract

The introduction in 2010 of the Freedom of Choice Act represents one of the most far-reaching reforms of the Swedish health system. While it is mandatory for the regional counties to introduce choice plans for primary care it is voluntary for ambulatory specialist services. The voluntary nature of the regulations for the latter types of care generates a potential gap between the central government’s reform attempts and the regional implementation of the plans. We review the regional implementation of this reform with respect to specialist services from a political economy perspective. Data on the scope of implementation show that counties of the same political ideology as the central government have introduced the most choice plans for specialist care. In particular, counties ruled by right-wing majorities have introduced the Choice Act to a considerably larger extent than left-wing counties. This creates a highly uneven situation across the various parts of the country, possibly at odds with the basic premises of the country’s health law of equal access to care. The introduction of choice plans forms part of a decidedly contentious set of issues that are high on the political agenda of Sweden. The nature and impacts of these reforms are also a concern to the general public and the broader industry. Considerably more rigorous analyses will be needed to assess the impact on key policy parameters such as overall system efficiency and equitable access to services as a result of these changes to the health care markets.

## Introduction

In 2008, the Swedish parliament passed a national law on freedom of choice by citizens across a number of sectors, including health and social care. The Freedom of Choice Act (law 2008:962; LOV) took effect in January 2009 and in many ways represents the most wide-sweeping changes to the Swedish health care system in many years. As most care is funded by regional taxes, the Choice Act meant that the law on public procurement (law 2007:1091; LOU) was replaced by the LOV in these sectors. In parallel to the Choice Act, an amendment to the existing national health act (Law 1982:763) was made that, from 2010, mandated the 21 regional counties to allow people to choose their primary health care provider and to allow private providers of such care to freely establish practices if they meet certain defined standards. The main objectives of the choice reform were to enhance citizen choice, expand the provision of private health care, and strengthen quality based competition between providers [1]. Policy makers also expected new innovations in models of care with more variations across business models.

With respect to ambulatory specialist health care (e.g. dermatology, ophthalmology, and physiotherapy) the decision on the part of the counties to introduce a choice plan for such services is voluntary. If, however, such a decision is made, the LOV regulations then apply. Among other things the legislation stipulates that when a choice plan is introduced an announcement needs to be made on a special platform on the internet called the freedom of choice web (www.valfrihetswebben.se). The decision to go ahead with establishing freedom of choice for specialist services is made by the respective county councils. These regional governing boards are made up of political party representatives elected in separate elections from that of the national parliament (and of the 290 municipality councils).^1^ In combination with the relatively far-reaching political and administrative autonomy of the lower levels of administration of the country, this creates a potential gap between the central government’s ambitions to introduce national health reform and the regional councils’ willingness to implement the reform. While the decision to introduce a choice plan for specialist medical care may vary across counties depending on local contexts any indication of variation due purely to political reasons may generate undesirable differences in access and utilization of medical services between different parts of the country.

The Freedom of Choice Act and the associated amendment to the existing health act built on the experiences of past regional reform efforts where citizens in some counties had been allowed to freely choose their primary care provider. The partially mandatory nature of the reform represents a relatively strong central government intervention in an area that is the responsibility of the regional county governments. It is also a reflection of the then center-right central government’s wish to expand freedom of patient choice and private provision of health services to the entire population in a sector that has traditionally been largely government run with limited patient ability to choose provider [2–4].

As the implementation of the Freedom of Choice Act continues it is of general interest to understand what drives the scope and pace of the new regulations. It is particularly relevant to assess the extent to which the reforms are guided by real policy concerns or if the forces of application are affected by the politically motivated ideologies on the role of the market versus the state in health care. This article reviews the implementation of the LOV as it relates to ambulatory specialist medical services. It specifically relates the regional implementation to the political situation in the particular county to obtain an understanding of the extent to which politics may be a strong factor in this process.

## Review

**Fig. 1:**
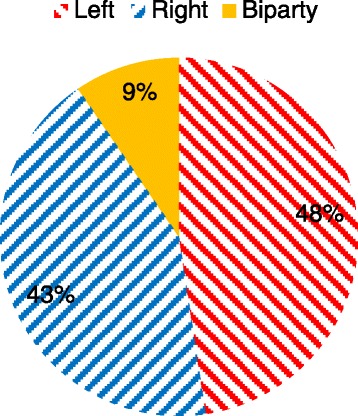
Political majority blocks, share of counties, post-election 2010. Sources: Swedish Association of Local Authorities and Regions, SKL

In the elections of 2010, the alliance of center-right parties (the Moderates, the Liberals, the Center Party, and the Christian Democrats) retained their parliamentary majority and was able to form the central government for a second four-year term. At the county level the elections generated a relatively balanced situation with ten left-wing majorities, nine center-right majorities, and two bipartisan coalitions of right- and left-wing parties (Fig. 1).

All else equal, the fairly even distribution between right-wing and left-wing majority councils would suggest that the scope of implementation of the LOV be comparatively uniform across the counties. However, the adoption of the new act is likely determined by contextual considerations, which may vary across counties.

### Implementation of reform

The implementation of the freedom of choice act is monitored by, among others, the Association of Local Authorities and Regions (SKL; a political organization working to advance the interests of the regional and local governments). The SKL presents data on the number of announcements for specialist health services that each county reports to the freedom of choice web portal. These data are combined with the information on the political majority of each county after the 2010 elections to assess the association between these two variables.

**Fig. 2:**
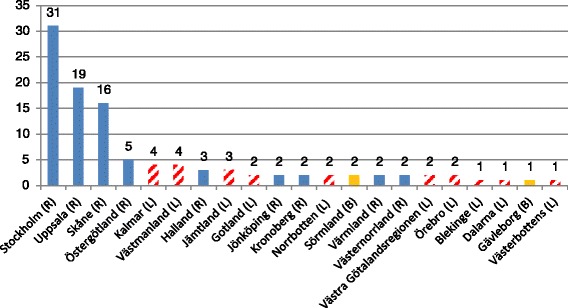
Implementation of LOV, December 2014. Implementation of the Freedom of Choice Act for ambulatory specialist care, December 2014. (L) Left-wing; (R) Right-wing; (B) Bipartisan. Source: Swedish Association of Local Authorities and Regions, SKL

As of December 2014, the most recently available data on the implementation of the freedom of choice act in specialist care, four of the fastest implementers of the LOV are all of the same political majority as that of the central government which introduced the act (R in Fig. 2). To date a total of 107 announcements have been made with a mean of 5.1 across all counties (up from 3.19 in June 2012 and 3.57 in May 2013, not shown).

Furthermore, the pace of the reform process is uneven across the counties. Three counties – Skåne, Stockholm, and Uppsala; all governed by center-right majorities – have implemented three to six times as many choice plans as the fourth largest county (Östergötland, also a center-right majority county). The counties that have implemented the Choice Act in this sector the least are all governed by left-wing majorities, or a Bipartisan coalition. In addition, more than three-quarters of all choice plans for specialist services had been introduced by counties with a center-right political majority that make up less than fifty percent of all counties (not shown). Around one-fifth of plans had been introduced by a left-wing led county (48 percent of all counties) and less than three percent by a bipartisan coalition.

The above analysis does not present conclusive evidence that the implementation of the Freedom of Choice Act as it relates to specialized health care in Sweden is solely driven by politics. The variation in the pace of implementation is most likely explained by several factors, both political and institutional. For example, it may be difficult to establish a competitive market for certain specialist services in some areas. These factors need to be controlled for in order to provide a more certain conclusion. However, the findings are fairly compelling and they are in line with those of studies from other countries and sectors.

### Comparisons with other sectors and countries

Many countries have opted to decentralize the responsibility for health care to lower levels of government and administrations. Examples of such countries include India, Canada, Australia, and the U.S. In addition, many central governments in these and other countries have tried to introduce health care reform that would in various ways impose mandates on the regional authorities. The U.S. is a case in point. In 2010, the federal government introduced the Affordable Care Act (ACA), one key objective of which is to expand health insurance coverage in that country. For its implementation, the ACA relied to a very large degree on the states to, among other things, set up insurance exchanges and expand Medicaid (a state-federal health care program for the poor). In a supreme court decision of June 2012 the expansion of the Medicaid program became voluntary for the states [5]. As of 2015, 28 states had expanded the program, although in different ways. In this sense, the situation for the ACA is similar to that of the LOV in Sweden.

Evidence also shows that the implementation of the ACA has been strongly influenced by politics, such that the establishment of insurance exchanges and the expansion of Medicaid, as well as other features of the reform, have varied across the states [5, 6]. Generally, implementation of the Act has been more far-reaching in states on the east coast and the west coast and less so in the middle of the country [7]. This pattern is broadly in line with the political majority of the states where Democrats tend to rule in coastal states and Republicans in the central parts of the country [8]. For example, of the seven states that had implemented all three of the mechanisms analyzed in Feller et al. (ibid.), five were Democrat. And of the five states that had implemented nothing of the provisions, four were Republican (authors’ own calculations based on Feller et al. and data on the 2012 presidential election outcomes by state).

The health sector is not the only area where regional implementation of national reform initiatives seems to follow political lines. In a recent analysis of local level outsourcing of schools in Sweden, Elinder and Jordal [9] find that “the political color of the ruling majority [of municipalities] influences outsourcing.” (page 43). Furthermore, the data suggest that right-wing majorities are more in favor than left-wing majorities to outsource the provision of primary schooling to private providers. The authors find support for the Citizen Candidate model of political behavior (see references in Elinder and Jordal for details of this and other political behavioral models). In light of these results, the findings presented in this review are not surprising in that introduction of choice programs in health and the outsourcing of primary schooling in many ways form part of a coherent political agenda of many center-right parties in Sweden and in other countries.

### Recent developments and future reform prospects

In the elections of September 2014, the center-right Alliance ceded power in the national parliament to a left leaning minority coalition government of Social Democrats and the Green Party. To some extent the change of government has led to uncertainty as to the future of the Choice Act in its current form. In particular, a proposition by one of the left-wing parties went so far as to propose that the mandatory introduction of the free choice of primary health at the county level be repealed (Proposition 2014/15:15). Notably, in the round of consultation responses to the proposition seven counties objected to the proposition, four of which are right-wing ruled and eight counties agreed with the proposition, five of which are left-wing governed [10]. However, due to technical inadequacies raised by the Council on Legislation the proposition had to be withdrawn providing at least a temporary stay for this part of the Choice Act. In addition, the parliamentary committee on social affairs has issued a suggestion to the government to refrain from repealing the Freedom of Choice Act as it relates to primary health care [11].

The national association of private health care providers has repeatedly raised concerns about the prospect of making changes to the Freedom of Choice Act. The view of the providers is that the choice plans have led to improvements in the delivery of medical services and increased access to care for the patients [12]. Although no rigorous and comprehensive impact evaluation of the Freedom of Choice Act for health care at the national level has been carried out, other more descriptive analyses of the effects of the plans in particular counties have found some support for these views [13–16]. In particular, Beckman and Anell [13] find that utilization of primary care increased after the introduction of the reform, particularly among better off households. Glenngård et al. [15] find that people in three sample counties reported that they had made an active choice of provider in connection with the reform. However, they also note that people were generally fairly passive in their choice behavior and tended to choose a provider that they had previously been in contact with.

Furthermore, in a series of national household surveys, the general public has expressed broad satisfaction with the increased ability to freely choose health care provider under the Choice Act [17]. That the choice plans are a highly contentious part of the current Swedish health care reforms became clear with the publication of a recent report by the Swedish National Audit Office [18]. The report found that the introduction of the choice plans had led to an increase in the use of care by the better-off at the expense of the worse-off. It also reported that the expectation of new innovations in the private provision of services has not been realized. These claims were, however, criticized by other analysts as overly selective and unsubstantiated.^2^

The seemingly strong public support for the free choice plans in health care and the relatively positive findings of the assessments of the reform on quality and access suggest that the free choice reforms will remain in place over the coming years. A special review is currently looking at ways to regulate private profit-making in the publically funded social sectors and while some measures are expected from this exercise, the counties will continue to allow their citizens to freely choose their health care providers under some regulatory framework.

## Conclusions

The Freedom of Choice Act represents one of the largest reforms of the Swedish health care system. While the reform may contribute to enhancing the efficiency of the overall system, the uneven implementation of the associated choice plans for specialist health services across counties appear to be mostly motived by the variation in political majority of the county councils. Right-wing majorities in a small number of counties have implemented the free choice plans to a considerably larger extent than the majority of counties, most of which are left-wing run. The variation raises questions about some of the most fundamental aspects of the national health act, namely equal access to care and the prioritization of those most in need. Further analyses are needed to assess these and other aspects of the actual implementation of the freedom of choice reforms in Sweden, the findings of which will be of interest also to other reforming countries.

## Footnotes

1 Sweden holds elections to the national parliament, the regional councils, and the municipalities’ governing boards on the same day every four years; the most recent round of elections was in September of 2014.

2
http://www.dagensmedicin.se/artiklar/2014/11/26/debatt-riksrevisionens-granskning-alltfor-selektiv/; in Swedish.
